# A Plea for Broader Tendencies in Veterinary Instruction1The address of the President of the American Veterinary Medical Association, Detroit, Michigan, September 4, 1900.

**Published:** 1900-09

**Authors:** Leonard Pearson

**Affiliations:** Philadelphia, Pa.


### A PLEA FOR BROADER TENDENCIES IN VETERINARY INSTRUCTION.[1]

By Leonard Pearson, B.S., V.M.D.,
PHILADELPHIA, PA.

The past year has been one of great progress in our Association affairs and in the development of our profession. The schools are receiving more support and are improving. Laboratories for research work in comparative pathology are being improved and enlarged, and new ones have been established. Under the stimulus of increased demand for their services and higher rewards the veterinarians of the country have given more attention to their organizations, have strengthened them, and, by coöperation, advanced the status and the conditions governing their calling.

In so far as this applies to our own organization you will be told more by the Secretary—that vigilant, untiring officer who keeps in touch with all of the details of the work of this international society from year's end to year's end; planning, pleading, prodding as conditions require or as instructed by you. The Secretary's office has steadily grown in importance, and is now the great reservoir and mainspring of the official work of this organization. The amount of time and thought required to satisfactorily conduct the affairs of that office and the difficulties experienced in arranging for a meeting such as this, and in keeping up the correspondence with nearly four hundred members, can scarcely be appreciated by one who has not undertaken it. Do we compensate the Secretary sufficiently for the time he must take from his personal work and devote to our business? I believe that this question should be carefully considered.

Our organization is now stronger and its influences are broader and more potent than ever before. We will better appreciate the meaning of this if we recall some of the achievements of this body, as, for example, the success of its efforts in the direction of lengthening the courses of instruction in the veterinary schools. When this matter was taken up a few years ago but three veterinary colleges in the United States and one in Canada required three years' attendance. At this time there are but two colleges in this country and one in Canada that do not require three years' attendance, and

[1] The address of the President of the American Veterinary Medical Association, Detroit, Michigan, September 4, 1900.

this out of a total of sixteen. Moreover, the requirements for entrance are gradually being raised, and students better prepared for the study of the veterinary sciences are now being required and received. These great advances are in part a natural development, but there can be no doubt that they have been encouraged and hastened by this body.

Whatever change of sentiment there may be among the members of Congress and on the part of the public in regard to the proposition to confer the authority and dignity of rank upon veterinarians in the United States Army is due principally to a few members of this body working as members of special committees. Especial mention should be made of the faithful labors of Dr. D. E. Salmon and of Dr. R. S. Huidekoper. By the efforts of these gentlemen, aided most valiantly by the members of this Association and the veterinary profession at large, it was possible at the last session of Congress to make a greater advance in the matter of placing veterinary work in the army on a proper basis than has ever been possible before. The bill drafted by your committee has passed the Senate, has gained friends in the House of Representatives, and has secured the indorsement of the President. It now remains for us to continue to follow the leadership of our Committee on Army Legislation, to sustain and aid them in the continuation of their difficult work. If they shall fail this year to secure the enactment of this reform that the army needs so sorely we must not be discouraged, but continue the struggle until the military veterinarians are given commissions and are organized into a compact and serviceable corps, with a veterinarian at their head whose rank is in proportion to the magnitude and importance of the work he is required to control.

I believe that the veterinary profession of the United States is determined to have its dues in this matter, and is determined that the army shall have a veterinary equipment that will be a credit to this country. The ancient system now in vogue—that is the source of enormous losses to the tax-payers, that lessens the efficiency of the army and is an insult to the veterinary profession—must be supplanted, no matter how much time and effort are required to do it.

In the publication of veterinary literature this Association has always taken a prominent and leading part. The oldest veterinary periodical in this country was originally the journal of this Association. During recent years its annual proceedings have constituted most important contributions to our literature. These volumes reflect the thought, the attainments, the ideals and purposes of what is highest and best in our profession. It is fitting, therefore, that they they should appear in a form that is commensurate with all they represent.

In the encouragement and development of the public relation of veterinary work this Association has always taken a foremost position. This will be shown by reference to the resolutions adopted at our annual meetings. These resolutions have come to be looked upon as the authorized voice of the veterinary profession, and so it is important that they shall always be well considered, conservative, and safe.

The papers that have been read at the meetings of this Association and the discussions upon them have had the effect, in large measure, of solidifying and unifying the thought and efforts of veterinarians on questions of moment. The methods employed in controlling various infectious diseases and the organization of State Boards of Veterinary Medical Examiners are instances in point.

The development during the past decade of knowledge of disease and of effective methods of prevention and cure have been marvellous indeed. It is gratifying to consider that much of this advancement has been the work of comparative pathologists and of veterinarians. It is only quite recently that the advantages have been recognized that may be derived from the study of comparative pathology in relation to the development of the whole subject of disease, and especially to human diseases. In fact, scarcely any attention was paid to this subject until Bollinger explored the almost virgin field of animal pathology thirty years ago. Virchow and Birch-Hirschfeld also recognized the value of the broadening discipline and the new lights to be derived from the study of the diseases of animals. More recently attention has been forcibly drawn to this subject by the researches of Johne, Kitt, Nocard, Smith, Moore, and McFadyean. In bacteriology, a younger branch of pathology, students have not been bound by such rigid traditions, and so they have not drawn sharp lines between genera and confined their studies to the organisms that attack a single kind of animal. Many bacteriologists, following the example of the great leaders, Pasteur and Koch, have studied the bacterial diseases of useful animals.

The idea is gaining ground that disease must be studied as disease in the broadest sense possible, and not, as heretofore, as a morbid process confined to a part of the body of a single species of animal.

"Comparative pathology" already has a familiar sound, and many laboratories are being erected or equipped and enlarged to make special provision for this new development. This movement is evident not only in this country, but abroad as well, and notably in Germany. These laboratories are usually connected with medical schools or Government health departments. One result of this, it is hoped, will be to throw new light on many of the obscure points in the pathology of the diseases of animals and help to overcome the condition that Johne described in explaining why he did not finish his text-book on pathology that was announced ten years ago. He said: "The pathological institutes in human medicine are filled with young physicians who have sought and found in this field a subject for a thesis or are undertaking advanced work. This results, at least, in the accumulation of a plentiful amount of material that without too much labor can be sifted and worked over. Thus building-stones are made from which the master can erect a building. Of this, veterinary pathology has nothing. The teacher of veterinary pathology is not supplied with sufficient help, and has scarcely time to attend to the current work of his department, the post-mortems and courses and lectures, if he is thorough in them. There are no positions to strive for or other encouraging conditions to attract young veterinarians to provide building-stones for great text-books."

Since these words are true of the conditions in America even more than in Germany, we hail with joy any movement that promises to provide facilities for and to encourage workers in comparative pathology. In view, however, of the existence of these great laboratories and the numerons pressing demands on the public exchequer aud the funds of universities, it will become more difficult to establish special laboratories for veterinary pathology.

But it is not at all likely that this development will fail to receive a warm and sympathetic welcome. We must not be content with this betterment, however, nor should we lightly fall into the error of thinking that this is all of the veterinary sciences. We must not allow the other essential veterinary sciences to be neglected and fail to develop in proportion to their fellows and their own innate importance.

If comparative pathology is taken up by laboratories usually conducted by physicians and principally for the purpose of throwing light on the diseases of man, and if our veterinary teachers must go to this source for their facts, we must not allow all of the veterinary sciences to be humanized in the same way and developed as side issues to subjects that are conceived to be more important.

It is evident that our veterinary schools and veterinary departments of universities must always stand on independent bases if they are to properly fulfil their function. It will never do for them to become subsidiary departments incidental to schools of medicine, for example. If any arguments are needed to support this contention they may be found in the vast importance to the nation of the interests that are protected and fostered by veterinarians and in the histories of veterinary schools in other countries.

First, veterinarians guard the greatest income-producing property in the United States. Live-stock husbandry is the most profitable branch of our agriculture; it feeds more people and furnishes more homes than any other branch of manufacturing commerce or trade. It furnishes material for exports amounting to more than $200,000,000 per annum. It represents an investment of more than $2,000,000,000. The success of our agricultural population, comprising 58 per cent. of the people of the country, depends on their horses, cattle, sheep, swine, and poultry more than on any other factor.

Moreover, the butchers, milk dealers, creameries, woollen mills, tanneries, shoe factories, hat factories, and numberless other smaller interests and industries depend on animals and animal products. The protection and development of such interests as these are worthy of the most earnest attention of statesmen, scientists, and educators.

Second, the experiment has been tried in Europe of establishing veterinary chairs in universities and attempting to provide veterinary courses without independent faculties and the complete equipment of a veterinary school. Such experiments at Halle, Leipsic, and Giessen have failed. The successful work of investigation and instruction in the veterinary sciences in Europe is done in separate veterinary schools, as at Berlin, Dresden, Hanover, Alfort, London, and Vienna. There are, perhaps, three seeming exceptions to this rule. In Berne the veterinary school has during the past year been made a department of the university, but its separate plant and faculty are preserved. In Copenhagen and Budapesth the veterinary schools are associated with schools of agriculture, but each has its special equipment and faculty, and this union appears to be advantageous to both institutions.

If the veterinary sciences are dominated by comparative pathology there is danger that the point of view of the veterinarian will become narrow, and that he will not be competent to apply to the best advantage the knowledge of comparative pathology that he possesses. This statement may at first seem paradoxical, but my thought is that it is necessary for a veterinarian to be thoroughly familiar with animals in health before he can treat them successfully *or economically* when they are diseased. It is necessary to know the breeds, values, foods, methods of rearing, using, etc., in order to be in a position to discover and remove causes of disease or advise appropriate treatment. This idea was illustrated to me by Professor Dieckerhoff, who said of a certain eminent physiologist, a professor in a veterinary school: "He could teach veterinary physiology better if he could ride"—that is, if this great scientist had possessed some familiarity with animals he could have extended and applied his profound knowledge beyond the walls of his laboratory and lecture hall to animals in the stable and pasture. There is as much in the application of principles as in propounding them. It is not enough to have a set of tools; one must know how to use them.

In spite of the prevailing popular impression, the veterinarian is more than a doctor of diseased and maimed beasts; and he is himself responsible for the narrow bounds that circumscribe his field of labor in the popular mind. In this country the title "doctor" does not commonly convey the impression of a learned man, but to the plain person it signifies one who doctors. Now, veterinarians have insisted upon the title of doctor, whether conferred upon them by their college or not, and the result has been that they are commonly regarded as men whose sole business is to doctor animals, and this is considered the single object and the end of their technical equipment.

In other countries veterinarians do not covet the title of doctor, and it results that they are not regarded as members of the least important branch of a noble profession, but stand on footing of their own.

Partly on account of the narrowing effect of a useless title, and partly through failure to realize the importance of the subject, veterinarians have not cultivated to the extent they deserve what might be called the physiological phases of animal husbandry. With his knowledge of comparative anatomy, physiology, chemistry, foods, and the predisposing and exciting causes of disease the veterinarian should be the natural expert on conformation, selection for special purposes, breeding, rearing, feeding, etc. He should also be thoroughly informed on stable construction and other practical questions related to the maintenance and utilization of animals.

As expert landscape gardeners, civil engineers, and architects are consulted on the problems within their professions, so should veterinarians be consulted on the problems within their profession covering all phases of animal husbandry. Before this can occur largely it will be necessary for the public to relinquish the idea that veterinarians are useful only to patch up decrepit animals or to check the spread of disease, however important this may be, and be educated to regard them as experts in animal husbandry—as animal engineers.

It is also necessary that our schools shall devote more attention to these subjects. At present our knowledge of some parts of them is almost purely empirical; it remains for veterinarians to formulate it and place it on a scientific basis, whence it may advance. Most of the accurate work that has been done in these lines has been done by veterinarians, beginning with the illustrious Bourgelat; but there is an enormous field that is still untouched.

This Association can do much to broaden the scope and practice of the veterinarian. It can encourage the preparation and reading of papers relating to the subjects that are usually grouped under the title "Animal Husbandry." It can appoint committees to report on special problems coming under this head. It can recommend the amplification, the deepening and broadening of instruction in zoötechnical and allied subjects in the veterinary schools. When all this is done the veterinary profession will be in more direct touch and in closer sympathy with the stockman, and cannot fail to be of more service to the live-stock industry and to itself profit by these more intimate bonds.

This thought must have been in the minds of those who planned and organized the federal Bureau of Animal Industry. It is fortunate that this bureau is established on broad lines; that it is not the bureau of comparative pathology or the bureau of veterinary police, but that its purpose is to improve the animal industry in all of its departments and relations, and that its organic law is broad enough to permit this.

In the same way, so far as an individual may, each veterinarian should extend his knowledge and his practice and become an expert in animal engineering.

In dwelling upon this as a legitimate and desirable field of effort I have not wished to appear antagonistic to any work the profession or this Association may now have in hand. It is all important and should all be strengthened. It is your high duty as the representative body of the veterinary profession of North America—as the Veterinary Senate—to point the way of advance and to strengthen the hands of the laborers in the field.

